# Adolescent THC Treatment Does Not Potentiate the Behavioral Effects in Adulthood of Maternal Immune Activation

**DOI:** 10.3390/cells10123503

**Published:** 2021-12-11

**Authors:** Todd M. Stollenwerk, Cecilia J. Hillard

**Affiliations:** Neuroscience Research Center, Department of Pharmacology and Toxicology, Medical College of Wisconsin, Wauwatosa, WI 53226, USA; tstollenwerk@mcw.edu

**Keywords:** polyinosinic-polycytidylic acid, schizophrenia, amphetamine hyperlocomotion, prepulse inhibition, Morris water maze, fear conditioning

## Abstract

Both in utero exposure to maternal immune activation and cannabis use during adolescence have been associated with increased risk for the development of schizophrenia; however, whether these exposures exert synergistic effects on brain function is not known. In the present study, mild maternal immune activation (MIA) was elicited in mice with prenatal exposure to polyinosinic-polycytidylic acid (poly(I:C)), and ∆^9^-tetrahydrocannabinol (THC) was provided throughout adolescence in cereal (3 mg/kg/day for 5 days). Neither THC nor MIA pretreatments altered activity in assays used to characterize hyperdopaminergic states in adulthood: amphetamine hyperlocomotion and prepulse inhibition of the acoustic startle reflex. Adolescent THC treatment elicited deficits in spatial memory and enhanced spatial reversal learning in adult female mice in the Morris water maze, while exposure to MIA elicited female-specific deficits in fear extinction learning in adulthood. There were no effects in these assays in adult males, nor were there interactions between THC and MIA in adult females. While doses of poly(I:C) and THC were sufficient to elicit behavioral effects, particularly relating to cognitive performance in females, there was no evidence that adolescent THC exposure synergized with the risk imposed by MIA to worsen behavioral outcomes in adult mice of either sex.

## 1. Introduction

Schizophrenia is a psychiatric disease with a multifactorial neurodevelopmental etiology. While genetic factors contribute to the risk of schizophrenia, environmental risk factors during critical times of neurodevelopment, particularly the prenatal period and adolescence, contribute to the disease etiology [1].

Maternal immune activation (MIA) is a risk factor for the development of schizophrenia as well as other psychiatric disorders. Prenatal exposure to viral pathogens, such as the influenza virus, is associated with increased incidence of schizophrenia in adulthood. For example, epidemiological studies demonstrate increased rates of schizophrenia following influenza epidemics [2,3,4,5]. Additionally, prenatal exposure to other pathogens has been shown to be associated with schizophrenia [6,7]. The widespread infections of the coronavirus disease 2019 (COVID-19) pandemic increase the need to improve understanding of the effects of MIA on neurodevelopment in offspring.

Animal models of MIA have been used to study neuropsychiatric diseases with neurodevelopmental etiologies, including schizophrenia [8,9,10]. One of the most widely used MIA animal models involves gestational administration of polyinosinic-polycytidylic acid (poly(I:C)), a double-stranded RNA (dsRNA) that activates the innate immune system through TLR-3 signaling. In this model, the offspring of poly(I:C)-treated dams develop abnormal behaviors in adulthood, including impaired sensorimotor gating (the ability to filter out extraneous stimuli) and enhanced sensitivity to stimulants such as amphetamine [11]. These effects can be reversed by antipsychotic drugs, supporting the relevance of this model to human disease [12].

Adolescent cannabis use is another risk factor for schizophrenia. Specifically, individuals with heavy or frequent cannabis use during adolescence, those who initiate use at younger age, and those who use high ∆^9^-tetrahydrocannabinol (THC)-containing cannabis have a greater risk for developing schizophrenia [13,14]. THC, the intoxicating component of cannabis, is a partial agonist of the CB1 cannabinoid receptor (CB1R), a G protein-coupled receptor (GPCR) found abundantly in the brain. Rats exposed to THC in adolescence, but not adulthood, have impaired sensorimotor gating and deficits in social interaction [15]. Additionally, these rats displayed a subcortical hyperdopaminergic state [15], which has been shown to underlie the positive symptoms of schizophrenia [16].

Whether adolescent THC treatment can potentiate the effects of prenatal immune activation to increase risk for schizophrenia is not known. The purpose of the studies in this report was to examine the individual and combined effects of MIA and non-stressful adolescent THC exposure on schizophrenia-associated behaviors in adulthood in mice. The effect of these treatments on behaviors that are characteristic of hyperdopaminergic states, namely amphetamine hyperlocomotion and prepulse inhibition of the acoustic startle reflex, and behaviors that test cognitive performance, specifically the Morris water maze (MWM) and fear conditioning assays, were examined in mice of both sexes. The degree of MIA and the dose of THC were designed to be subthreshold to interrogate whether there are synergistic effects of these treatments. These results indicate that although the doses used elicited some behavioral effects on their own, there was no indication of synergism, particularly in behaviors that are relevant to hyperdopaminergic states.

## 2. Materials and Methods

### 2.1. Animals

**Figure 1:**
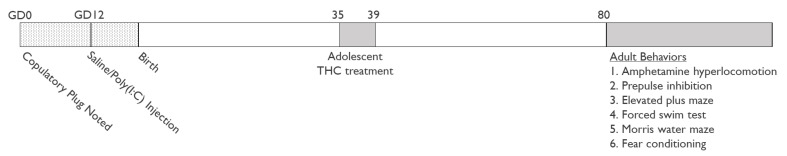
Experimental design.

Adult male and female C57BL/6J mice (6–12 weeks old) were purchased from The Jackson Laboratory (Stock number 000664, Bar Harbor, ME, USA) and were housed in individually ventilated cages in temperature- and humidity-controlled, specific pathogen-free facility with 12 h light/dark cycles (lights on 06:00 A.M.–06:00 P.M.) with food and water ad libitum. Breeders were acclimated to the facility for at least 1 week before placing 2 females in a cage with 1 male. Mice were checked daily for copulatory plugs, and gestational day (GD) 0 was ascribed to the first detection of a plug (Figure 1). All animal studies were approved by the Medical College of Wisconsin Institutional Animal Care and Use Committee prior to experimentation (animal use authorizations 00642 and 00141).

### 2.2. Drugs and Treatment

#### 2.2.1. Maternal Immune Activation

Polyinosinic-polycytidylic acid potassium salt was purchased from Sigma-Aldrich (St. Louis, MO, USA) and dissolved in saline at a concentration of 1 mg/mL. Poly(I:C) was administered to pregnant C57B6/J dams in the MIA group by intraperitoneal (i.p.) injection (5 mg/kg) on GD 12. The control group received an injection of the same volume of saline. Offspring were weaned 21–28 days after birth and housed in litter- and sex-specific groups of 2 mice per cage. One male and one female from each litter was used for each treatment group.

#### 2.2.2. THC Treatment

THC was the generous gift of the NIDA Drug Supply Program. THC was administered orally through voluntary consumption of a cereal piece (Corn Pops cereal) containing THC. THC (30 µL of 2 mg/mL in ethanol) or ethanol (30 µL) were added to the cereal using an insulin syringe with a 27 g needle. Ethanol was removed from cereal pieces by baking in a 40 °C oven for 40 min. Complete ethanol evaporation was verified by initial testing in which the mass of the cereal pre-injection, post-injection, and post-drying were compared. Complete consumption of the cereal piece results in the delivery of approximately 3 mg/kg THC.

The offspring of saline- and poly(I:C)-injected pregnant dams were individually housed on PND 35 and were fed cereal containing either THC or ethanol vehicle for five consecutive days. Each cage was inspected the following day for cereal remnants following treatment, and, if found, the mouse was removed from this study. On PND 40, mice were rehoused with their prior cage mates until behavioral testing beginning on PND 80.

### 2.3. Behavioral Assays

#### 2.3.1. Behavior in the Open Field and Amphetamine Hyperlocomotion

Mice were placed in an open-field arena (diameter 47 cm; height 33 cm) that was cleaned with 70% isopropanol between mice. Behavior was recorded with a ceiling-mounted Sony Handycam (HDR-CX405) and was analyzed by AnyMaze Behavior Tracking Software (Stoelting, Wood Dale, IL, USA).

Mice were habituated to the open field for 30 min. During this time, total distance traveled, time immobile, and time in the outer zone were measured. Mice were removed and injected with saline (4 mL/kg), and their movement was tracked for an additional 30 min. Finally, mice were injected with d-amphetamine (2.5 mg/kg, i.p., Sigma-Aldrich, St. Louis, MO, USA) dissolved in saline, and movement was tracked for 90 min. Locomotor activity was indexed as distance traveled, collected in 5 min bins. The data following the saline and d-amphetamine injections were analyzed separately.

#### 2.3.2. Prepulse Inhibition of the Acoustic Startle Reflex

Mice were tested in two startle chambers for mice (SOF-825, MED Associates, St. Albans, VT, USA). Following two minutes of acclimation in the apparatus, mice were exposed to a block of six startle pulses to allow for habituation and stabilization of startle responses. Mice were exposed to 12 blocks of test trials containing one of each of the following trial types: startle-alone, prepulse-plus-startle pulse at each of the five prepulse intensities, and no stimulus. The session concluded with a final block of six consecutive startle pulses. The startle pulse was a 40 ms pulse of 120 dB white noise. The background noise was a 65 dB white noise, and the prepulses were 20 ms bursts of white noise of the following intensities: 69, 73, 77, 81, and 85 dB (corresponding to 4, 8, 12, 16, and 20 dB above background). The interval between prepulse and startle pulse was 100 ms, and the intertrial interval was variable with a mean of 15 s (ranging from 10 to 20 s). The magnitude of PPI was calculated as [(pulse-alone)-(prepulse-plus-pulse)]/(pulse-alone) ×100%.

#### 2.3.3. Morris Water Maze (MWM)

The Morris water maze consisted of a pool (81 cm diameter) filled with water (22–24 °C). Non-toxic white acrylic paint was added to the water. An escape platform (7.5 cm × 7.5 cm) was located 20 cm from the side wall of the pool during acquisition and reversal learning. The platform was located 0.5 cm above the surface of the water on the first acquision day and was located 0.5 cm below the surface of the water for all subsequent days. Stable maze-external cues on the walls of the testing room allowed for spatial orientation.

Mice were subjected to four acquisition sessions on consecutive days. Each session consisted of four trials in which mice were placed into each of the four starting positions (north, south, east, west) in a random order. Animals were placed into the water facing the wall of the pool and were allowed to search for the platform for up to 60 s. Latency to find the platform was measured for each trial. If the mouse did not find the platform position within 60 s, it was guided to the platform position.

On the fifth day, a probe trial was performed in which the platform was removed, and the movement of the mice was tracked for 60 s. During the probe trial, AnyMaze software was used to measure entries into the platform area, time in the correct quadrant, and distance from the platform center.

On the sixth day, the platform was moved to the opposite quadrant of the pool, and mice were subjected to four trials, once from each starting position, to locate the new platform position. Latency to find the new platform location was measured for each trial. The average of the final three trials was reported.

#### 2.3.4. Fear Conditioning and Extinction

For fear conditioning, the protocol used was similar to that described previously [17]. A standard conditioning mouse chamber (30 cm × 30 cm × 25 cm) (Actimetrics, Wilmette, IL, USA) within a sound- and light-attenuating box illuminated by one 25 W bulb was used. The experimental contigencies were controlled by a computer via FreezeFrame software (Actimetrics), which also analyzed freezing behavior.

On day 1, mice were placed in the fear conditioning chamber. After 180 s of habituation to the chamber, they were presented with seven conditioned stimulus–unconditioned stimulus (CS–US) pairings (i.e., auditory tone and footshock) separated by 200 s. Each tone (80 dB, 420 Hz) lasted 20 s. During the last 2 s of the tone, a 0.5 mA footshock was delivered through a grid floor.

On the following five days, a fear extinction protocol was performed. Mice were tested in a novel environment where the walls and floor of the fear conditioning chamber were covered with black plastic surfaces sprayed with 1% acetic acid and in which the illuminating light was red. Mice were exposed to 20 tones (80 dB, 420 Hz) with durations of 20 s, separated by 200 s, without foot shocks. In all sessions, percent freezing time during the tone was measured. The average percent time freezing during the first 5 tones in each extinction session was reported.

#### 2.3.5. Elevated Plus Maze

Mice were placed on an apparatus with two opposite open arms (30 cm × 5 cm) and two enclosed arms (30 cm × 5cm with 15 cm walls) elevated 40 cm from the floor. They were allowed to explore the maze for five minutes, during which time their movement was recorded by the camera described above and analyzed by AnyMaze software. Times in the open and closed arms were measured and used to calculate a ratio of time in the open arms to time in the closed arms.

#### 2.3.6. Forced Swim Test

Mice were placed into a beaker of water (22–24 °C) for 6 min. During this time, movement of the mouse was recorded with a camera. The time that the mice spent struggling to escape versus floating or swimming was measured.

### 2.4. Statistical Analyses

Data were analyzed by three-way ANOVA, using the Geisser–Greenhouse correction to adjust for a lack of sphericity for repeated-measures. Post hoc analysis using the Šídák’s multiple comparisons test was performed when appropriate. Statistical outliers were determined using the ROUT method with Q = 1% and described when found.

## 3. Results

### 3.1. Amphetamine Hyperlocomotion

**Figure 2:**
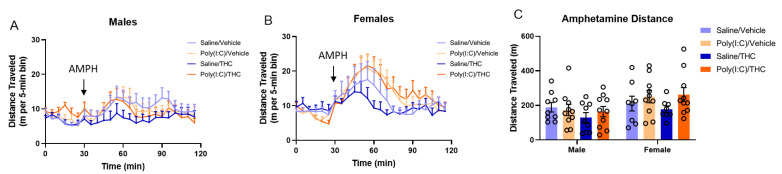
Amphetamine hyperlocomotion. (**A**,**B**) Distance traveled by male (**A**) and female (**B**) mice following saline (0–30 min) and d-amphetamine (AMPH, 2.5 mg/kg, 30–120 min) reported in 5 min bins. (**C**) Total distance traveled by mice during the 90 min following d-amphetamine injection. One female in the saline/THC group was removed from analysis as a statistical outlier (Rout, Q = 1%). Bar height represents the mean, vertical lines the SEM.

An enhanced locomotor reaction to low doses of amphetamine is interpreted as a functional imbalance in mesolimbic dopaminergic transmission [11]. The effects of prenatal poly(I:C) and adolescent THC treatment alone and in combination on the locomotor response to d-amphetamine (2.5 mg/kg) were determined in adult male and female offspring. In male offspring (Figure 2A), three-way repeated-measures ANOVA of the distance traveled time course data demonstrated that neither poly(I:C) (F_(1,34)_ = 0.09, *p* = 0.77) nor THC (F_(1,34)_ = 1.28, *p* = 0.27) had significant effects alone, and their interaction was not significant (F_(1,34)_ = 0.70, *p* = 0.27). As expected, there was a significant effect of time after amphetamine treatment (F_(3.411,116)_ = 3.34, *p* < 0.05). In female offspring (Figure 2B), three-way repeated-measures ANOVA of the distance traveled time course data demonstrated a trend towards a main effect of poly(I:C) (F_(1,32)_ = 2.92, *p* = 0.10) and no effect of THC (F_(1,32)_ = 0.08, *p* = 0.78) with a significant main effect of time after amphetamine treatment (F_(2.708,86.66)_ = 9.25, *p* < 0.0001). There was a significant time × poly(I:C) interaction (F_(17,544)_ = 1.82, *p* < 0.05); inspection of the data indicates that poly(I:C) treatment tended to increase distance travelled around the peak time. There was no poly(I:C) × THC interaction (F_(1,32)_ = 0.38, *p* = 0.54). When the total distance traveled in the 90 min following injection of amphetamine (Figure 2C) was analyzed by three-way ANOVA (with sex as the third factor), there were no effects of poly(I:C) (F_(1,66)_ = 2.31, *p* = 0.13) nor THC (F_(1,66)_ = 0.89, *p* = 0.35) and no poly(I:C) × THC interaction (F_(1,66)_ = 1.03, *p* = 0.31); however, there was a significant effect of sex (F_(1,66)_ = 6.73, *p* < 0.05), with males showing a decreased locomotor response to amphetamine compared to females.

The total distance traveled during the first 30 min of placement into the open field was used as a measurement of the effects of the treatments on baseline locomotor activity. Similarly, the total distance traveled following saline injection is a measure of acclimation to the open field and response to a mild stress. There were no differences in baseline locomotor activity (Figure S1) or in total distance travelled in the 30 min following saline injection (Figure S2) among the treatment groups.

### 3.2. Prepulse Inhibition of the Acoustic Startle Response

The second assay used to evaluate behaviors associated with hyperdopaminergic states is prepulse inhibition (PPI), a test of sensorimotor gating that assesses the ability of a prepulse to inhibit the startle response to a strong acoustic stimulus. Deficits in PPI have been consistently reported in patients with schizophrenia and have been observed in animal models of schizophrenia [18].

**Figure 3:**
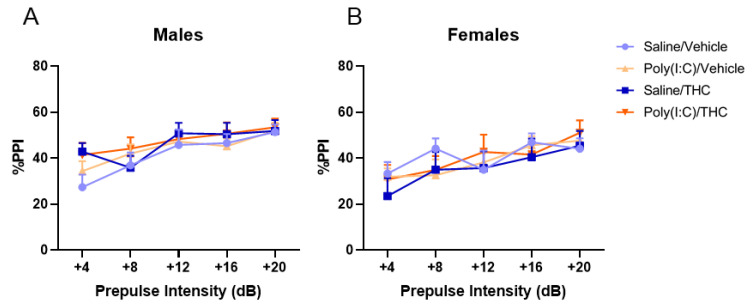
Prepulse inhibition (PPI) of the acoustic startle reflex in male (**A**) and female (**B**) adult offspring. The average percent PPI at five prepulse intensities was calculated. There were no effects of poly(I:C) or THC on PPI. All values are the mean ± SEM. *n* = 8–10 mice/sex/treatment.

The effects of prenatal poly(I:C) and adolescent THC treatment on prepulse inhibition of the acoustic startle reflex in adulthood were examined in male and female mice (Figure 3). As expected, there was a significant main effect of prepulse intensity for both male (F_(3.231, 109.8)_ = 16.53, *p* < 0.0001) and female (F_(3.375,111.4)_ = 14.71, *p* < 0.0001) mice. However, there were no significant effects of either poly(I:C) (male: F_(1,34)_ = 0.30, *p* = 0.59; female: F_(1,33)_ = 0.07, *p* = 0.80) or THC (male: F_(1,34)_ = 1.39, *p* = 0.25; female: F_(1,33)_ = 0.12, *p* = 0.73) treatments and no significant poly(I:C) × THC interaction (male: F_(1,34)_ = 0.04, *p* = 0.85; female: F_(1,33)_ = 0.30, *p* = 0.59). Startle amplitude was not affected by either treatment alone or their combination (Figure S3).

### 3.3. Morris Water Maze

**Figure 4:**
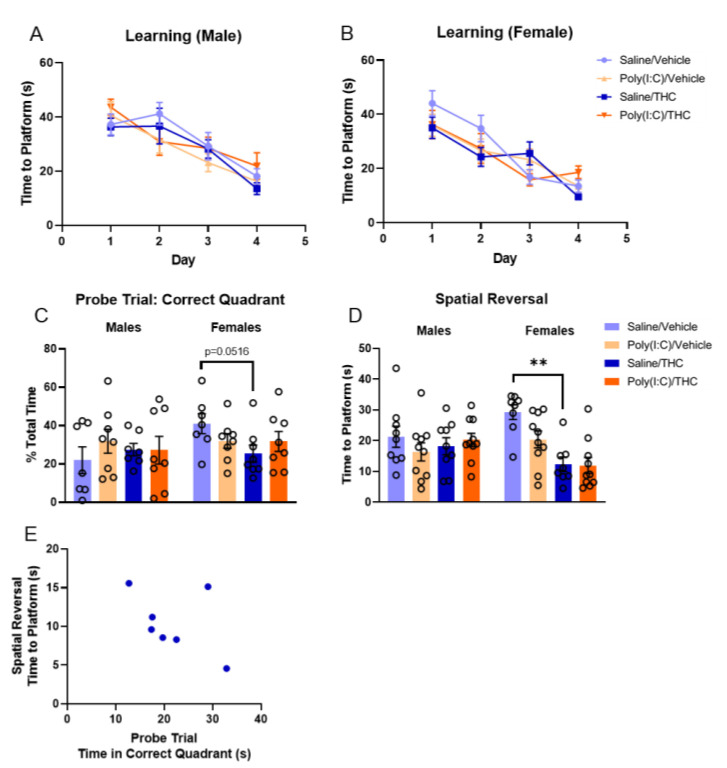
Morris water maze (MWM). (**A**,**B**) The average time that it took male (**A**) and female (**B**) mice to reach the platform during the training phase. The average of the four trials from each day was reported. (**C**) The percent of total time that mice spent in the correct quadrant of the MWM during the probe trial when the platform was removed. (**D**) The average time that it took mice to find the new platform location when the platform was moved during the spatial reversal phase. The average of the last three trials was reported. One female mouse in the saline/THC group was excluded from analysis as a statistical outlier (ROUT, Q = 1%). (**E**) Correlation between time spent in the correct quadrant in the probe trial and the average time to platform in the spatial reversal trial for female mice treated with saline prenatally and adolescent THC. Except for panel E, all bars represent mean and vertical lines the SEM. ** *p* < 0.01, based on analysis with Šídák’s multiple comparisons post hoc test.

The Morris water maze (MWM) was used to evaluate spatial learning and memory in adulthood following prenatal poly(I:C) and adolescent THC treatments. During the first four days of the assay, mice learned to find a submerged platform (Figure 4A,B). The effects of each treatment on the time to find the platform over training days were separately analyzed in male and female mice using a three-way repeated-measures ANOVA. In male mice, neither poly(I:C) (F_(1,34)_ = 0.001, *p* = 0.97) nor THC (F_(1,34)_ = 0.01, *p* = 0.92) had significant effects on the MWM behavior; the interaction between the treatments was also not significant (F_(1,34)_ = 0.631, *p* = 0.43). There was a significant day × poly(I:C) interaction (F_(3,102)_ = 3.61, *p* < 0.05) in the male mice; and, although post hoc tests were insignificant, inspection of the data suggests this is driven by a greater improvement in the time to find the submerged platform between days 1 and 2 for poly(I:C)-treated mice compared to saline controls. As expected, there was a significant main effect of day (F_(3,102)_ = 28.65, *p* < 0.0001). For female mice, neither poly(I:C) (F_(1,33)_ = 0.30, *p* = 0.59) nor THC (F_(1,33)_ = 0.21, *p* = 0.65) had significant effects alone. However, there was a significant day × poly(I:C) × THC interaction (F_(3,99)_ = 3.65, *p* < 0.05); although post hoc tests were not significant, those in the saline/vehicle group tended to perform worse initially. There was a significant main effect of day (F_(2.615,86.29)_ = 36.51, *p* < 0.0001).

On the fifth day, a probe trial was performed in which the platform was removed, and the movement of the mice was tracked (Figure 4C). Time that the mice spent in the correct quadrant was analyzed by three-way ANOVA (with sex as the third factor). There were no significant main effects of poly(I:C) (F_(1,53)_ = 0.20, *p* = 0.66), THC (F_(1,53)_ = 0.92, *p* = 0.34), or sex (F_(1,53)_ = 1.9, *p* = 0.17); however, there was a trend towards a sex × poly(I:C) × THC interaction (F_(1,53)_ = 2.70, *p* = 0.11). Post hoc tests indicate that THC treatment resulted in a trend towards decreased time in the correct quadrant in female mice treated with saline prenatally (Fisher’s LSD, *p* = 0.0516). No effects of any treatment were seen in male mice during this probe trial.

On the sixth day, the platform was moved to a new quadrant and latency to reach the platform was measured (Figure 4D). When the data for all mice were analyzed by three-way ANOVA, there were no significant main effects of poly(I:C) (F_(1,66)_ = 2.49, *p* = 0.12) or sex (F_(1,66)_ = 0.072, *p* = 0.79). However, there was a significant main effect of THC (F_(1,66)_ = 10.15, *p* < 0.01), a significant sex × THC interaction (F_(1,66)_ = 11.50, *p* < 0.01), and a significant poly(I:C) × THC interaction (F_(1,66)_ = 3.987, *p* < 0.05). Post hoc analysis with Šídák’s multiple comparisons test showed a significant difference in the adolescent effects of THC and vehicle in female mice who were treated with saline prenatally (*p* < 0.01). This indicates that female mice that were treated with adolescent THC were able to learn the new location of the platform more quickly than vehicle-treated mice. There were no effects of any treatment on the ability of male mice to learn the new platform location. While not statistically significant, there was a trend towards a negative correlation between performance in the probe trial and performance in the spatial reversal trial for saline-treated female mice given adolescent THC (Figure 4E, Pearson *r* = −0.45, *p* = 0.31). There were no similar correlations seen in the other treatment groups. This suggests that improved performance during the spatial reversal trial may be due, in part, to impaired memory of the platform location from previous days with intact short-term memory for learning during within-session trials.

### 3.4. Fear Conditioning and Extinction

**Figure 5:**
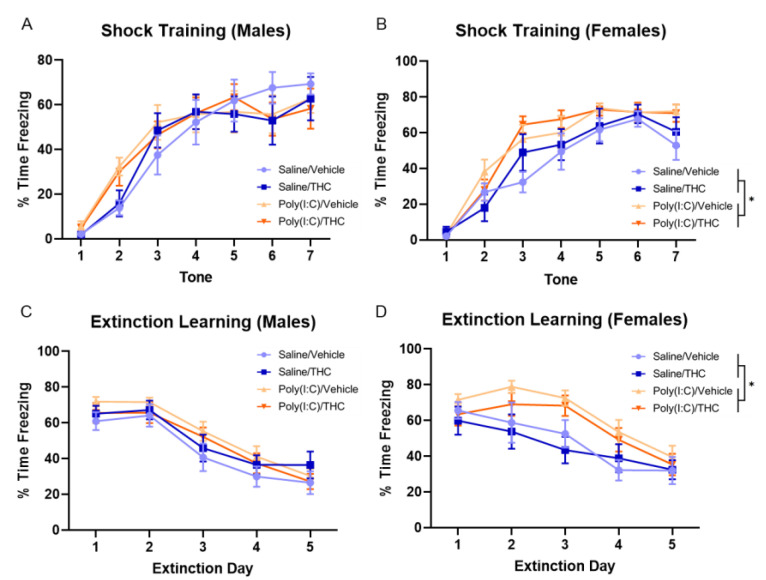
Fear conditioning and extinction. (**A**,**B**) The percent of time that male (**A**) and female (**B**) mice spent freezing during the tone in the training phase where mice received a foot shock at the end of the tone. (**C**,**D**). The percent of time that male (**C**) and female (**D**) mice spent freezing during the first five tones on each day the extinction phase. The data for four female mice on day two and two female mice on day five were lost due to technological failures. All values are the mean ± SEM. * *p* < 0.05, based on three-way repeated-measures ANOVA. *n* = 8–10 mice/sex/treatment.

Fear conditioning and extinction were used to examine Pavlovian learning and memory in adulthood in the treatment groups. On the first day, mice were exposed to seven tones paired with foot shocks. The learning of this pairing is represented by an increase in the time spent freezing during the tone. When freezing to each tone presentation was analyzed by a three-way repeated-measures ANOVA, there was a main effect of tone presentation for both male (F_(4.014,136.5)_ = 66.62, *p* < 0.0001) and female (F_(4.610,47.5)_ = 90.74, *p* < 0.0001) mice, indicating that animals were able to learn to associate the tone with an impending foot shock. For male mice, there were no significant effects of poly(I:C) (F_(1,34)_ = 0.25, *p* = 0.62) or THC (F_(1,34)_ = 0.066, *p* = 0.80) or a poly(I:C) × THC interaction (F_(1,34)_ = 0.0005, *p* = 0.98), but there was a trend towards a tone × poly(I:C) interaction (F_(6,204)_ = 1.89, *p* = 0.08) (Figure 5A). When the behavior of female mice was analyzed, there was a significant effect of poly(I:C) (F_(1,32)_ = 6.96, *p* < 0.05) but not THC (F_(1,32)_ = 0.31, *p* = 0.58), and there was no poly(I:C) × THC interaction (F_(1,34)_ = 0.20, *p* = 0.66) (Figure 5B). These results indicate that female mice exposed to prenatal poly(I:C) treatment learned to associate the tone with the shock more quickly than saline-treated mice.

On the following five days, mice were exposed to tones without foot shocks, resulting in decreased freezing over time as the mice learn that the tone is no longer associated with a foot shock. When the freezing behavior over the extinction learning days was analyzed by three-way repeated-measures ANOVA separately for male and female mice, there was a significant effect of day for both male (F_(2.706,92.01)_ = 71.20, *p* < 0.0001) and female (F_(4,122)_ = 29.02, *p* < 0.0001) mice. For male mice, there were no significant effects of poly(I:C) (F_(1,34)_ = 1.12, *p* = 0.30) or THC (F_(1,34)_ = 0.026, *p* = 0.87), and there was no poly(I:C) × THC interaction (F_(1,34)_ = 1.48, *p* = 0.23) (Figure 5C). For female mice, there was a significant effect of poly(I:C) (F_(1,32)_ = 8.34, *p* < 0.01) but not THC (F_(1,32)_ = 0.87, *p* = 0.36), and there was no poly(I:C) × THC interaction (F_(1,34)_ = 0.09, *p* = 0.77) (Figure 5D). These findings indicate that female, but not male, mice treated with poly(I:C) prenatally had slower extinction learning compared with those treated with saline.

### 3.5. Elevated Plus Maze and Forced Swim Test

There were no effects of poly(I:C) or THC treatment on elevated plus maze performance (Figure S4) or behavior in the forced swim test (Figure S5).

## 4. Discussion

There is no evidence in this study that MIA and adolescent THC exposure synergistically interact to cause behavioral deficits in adulthood. Doses of poly(I:C) (5 mg/kg) and THC (3 mg/kg) were used that would elicit mild effects yet allow for the identification of synergism without reaching a ceiling effect. While both treatments were sufficient to elicit some behavioral effects in adulthood at the doses used, particularly in female mice, we cannot rule out the possibility that synergistic effects would occur if higher doses were used.

We found that female, but not male, mice exposed to MIA had enhanced hyperlocomotion following amphetamine. More robust enhancement of amphetamine-induced hyperlocomotion by MIA has been shown in previous studies without a sex effect [11,19,20]. The small effect size observed in the current study is likely due to the use of intraperitoneal administration of poly(I:C) compared to the intravenous administration used in previous studies. It is possible that female mice are more sensitive than male mice to the lower level of MIA achieved in this study. Alternatively, all of the mice were exposed to a period of social isolation during adolescence, which likely has a greater impact on female adolescent mice [21]. Importantly, stress during adolescence has been shown to synergize with subthreshold MIA to induce behavioral deficits in adulthood [22].

PPI deficits have been shown in patients with schizophrenia as well as in animal models of schizophrenia [18]. Specifically, deficits in PPI in humans with schizophrenia have been shown to be associated with an inability to filter out irrelevant stimuli from the environment. In this study, there were no differences in PPI performance following either prenatal poly(I:C) or adolescent THC treatment. Although other groups have reported PPI deficits in rodents treated with prenatal poly(I:C) [11,23], these effects have not been universally demonstrated [24,25]. The origin of breeders [26] and the timing and dosing of poly(I:C) [19] have been found to affect PPI response. Although we used the same dose and timing as others who reported PPI (5 mg/kg dose at GD12), we employed intraperitoneal rather than intravenous injections to achieve a subthreshold inflammatory state [27,28].

Cognitive symptoms contribute substantially to the disease burden caused by schizophrenia. Individuals with schizophrenia perform about two standard deviations below healthy controls in neurocognitive tests [29,30]. Cognitive symptoms are better able to explain the poor functional outcomes, such as work performance and independent living, than positive or negative symptoms [31]. In this study, sex-specific effects of MIA and adolescent THC exposure were seen in both cognitive domains examined.

Female mice exposed to prenatal poly(I:C) exhibited more rapid learning of the freezing response during fear conditioning but had impaired extinction of the cued conditioned response. These effects were not seen in male mice. While several studies have found variable effects of MIA on the acquisition of conditioned stimulus-cued fear learning and memory [32,33,34], few studies have examined the effects of MIA on fear extinction learning. Two studies failed to find effects of prenatal poly(I:C) treatment on fear extinction [35,36]; however, in these studies, the length of extinction trials was limited to a single day or two days with shortened protocols. These findings are consistent with the results of this study in that the deficits in fear extinction were not immediately evident.

In the MWM, female mice treated with THC had a trend toward impaired spatial retrieval memory but had significantly enhanced spatial reversal learning. These effects were not seen in male mice. Previous studies have shown mixed results when exploring the effects of MIA and adolescent THC exposure on MWM performance. While some studies have shown impaired learning and memory with MIA treatment [23,37], other studies failed to show any impairment [38,39]. Likewise, some studies have shown deficits in MWM performance in adulthood following adolescent THC exposure [40], while other studies have failed to show an effect of adolescent THC treatment [41,42].

Our finding of enhanced spatial reversal, representing an improved ability to learn the new location, in female mice exposed to adolescent THC is surprising. While this could be potentially explained by a change in the exploration pattern, allowing for more rapid surveying of the pool area with less reliance on memory of the platform location, our data suggest that this is not the case. Instead, it is likely that the improved performance may be due to a relative preservation of short-term learning in the setting of impaired memory of the platform position from previous days. This would allow more rapid learning of the platform location on the spatial reversal day without a strong contradicting memory of a previous platform location, giving the appearance of improved cognitive flexibility.

The sex-specific effect of adolescent THC in MWM performance could be the result of sex differences in the metabolism of THC, with females showing a greater concentrations of the active THC metabolite (11-OH-THC) in both the plasma and brain [43]. A recent study also showed that there is a lower brain-to-plasma ratio of THC in adolescent male mice compared to adults [44], suggesting that the brains of adolescent male mice may be relatively protected from the effects of THC.

The fact that effects of MIA and THC were seen in the fear conditioning and MWM assays, respectively, indicates that the doses of prenatal poly(I:C) and adolescent THC were sufficient to elicit changes in cognitive performance in adult female mice. These data indicate that the doses that were chosen for this study can produce pharmacological effects. However, even in cognitive domains where individual effects of these treatments were seen, there were no interactions between these treatments.

A limitation of this study is that, although we checked cages to ensure that the entire cereal pieces were eaten, we cannot be certain that all mice received the entire 3 mg/kg THC dose. Studies to test this, such as measuring blood concentrations of THC or measuring changes in body temperature, would be complicated by the ad libitum ingestion of cereal. Additionally, these measurements would introduce stress that was intentionally avoided by providing voluntary oral dosing of THC.

While epidemiological studies have repeatedly found that adolescent cannabis use is associated with increased odds for developing schizophrenia [13,14], the causal relationship of this association remains unclear. A recent study examined the effect of adolescent cannabis use and cannabis use disorder symptoms on psychosis proneness in adulthood in two cohorts of twins [45]. This study found that cumulative cannabis use and the presence of cannabis use disorder were associated with higher scores of psychosis proneness in adulthood. However, in co-twin controlled models, which compared the greater-cannabis-using twin to the lesser-using twin, there was no effect of cannabis use on psychosis proneness. The study concluded that the association is more likely attributable to familial confounds rather than a direct causal effect of cannabis exposure. These findings are generally in line with previous twin studies [46,47], which suggest that the association between adolescent cannabis use and psychosis in adulthood is indirect and the result of environmental and genetic factors that contribute independently to risk for cannabis use and psychosis.

The findings in human studies correspond well with recent findings in preclinical models. For example, adolescent administration of THC in rats attenuated the effects of MIA on dopaminergic neuron firing in the ventral tegmental area (VTA) rather than worsening the MIA-induced deficits [48]. This suggests that the interaction between adolescent THC and MIA is not straightforward, particularly in relation to dopaminergic signaling in the brain.

Future directions for this research include further exploration of the effects of adolescent THC exposure on reversal learning, including determining whether the effect of adolescent THC to induce enhanced spatial reversal performance is due to impaired memory or enhanced cognitive flexibility. Additionally, the effects of adolescent THC exposure on adult behavior should be investigated in other models of schizophrenia risk to determine whether these findings are generalizable.

The current study does not support the hypothesis that adolescent exposure to THC interacts with the risk associated with MIA to synergistically worsen behavioral outcomes in adult mice. While it is possible that adolescent THC exposure interacts with other genetic or environmental risk factors to increase risk for schizophrenia, our study does not support a direct etiological role of adolescent THC exposure in the development of schizophrenia.

## Data Availability

Authors will provide raw data upon request.

## Supplementary Materials

The following are available online at https://www.mdpi.com/article/10.3390/cells10123503/s1, Figure S1: Open-field behavior; Figure S2: Distance traveled following saline administration in the amphetamine hyperlocomotion assay; Figure S3: Startle amplitude in the PPI assay; Figure S4: Elevated plus maze; Figure S5: Forced swim test.
