# Supplementary material for: Adolescent THC Treatment Does Not Potentiate the Behavioral Effects in Adulthood of Maternal Immune Activation
Source: Cells. 2021 Dec 11;10(12):3503. doi: 10.3390/cells10123503 (PMC8700174; doi:10.3390/cells10123503)
Supplement: Supplementary file 1 [file cells-10-03503-s001.zip › cells-1488396-supplementary.pdf]

Supplementary Data

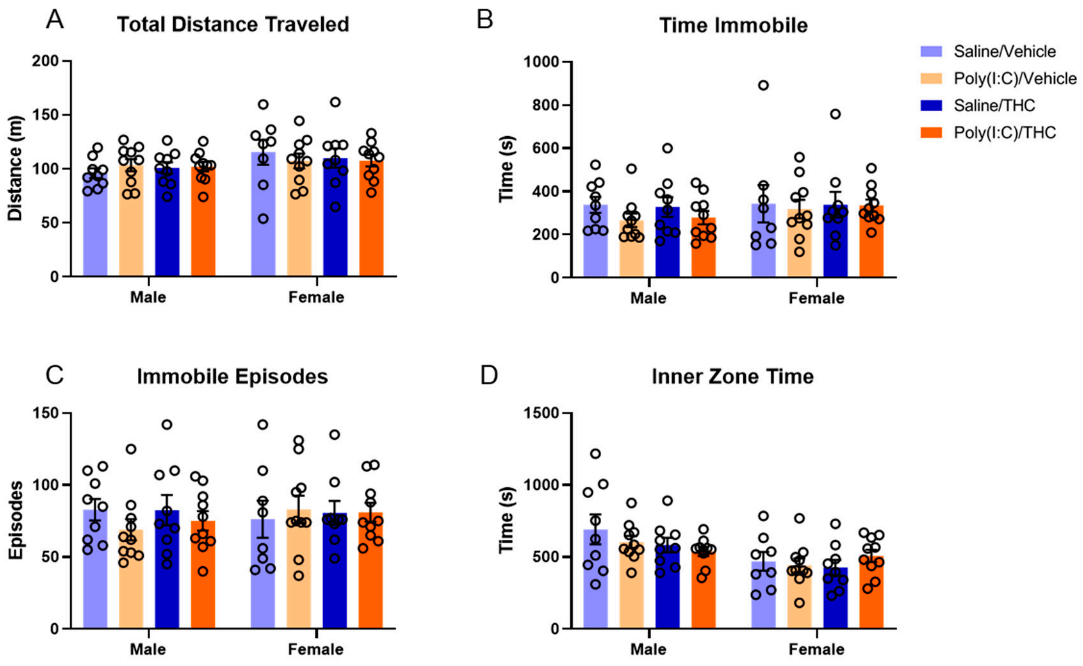

Figure S1. Open field behavior during the first 30 minutes that mice were placed in the open field prior to the amphetamine hyperlocomotion assay. (**A**) Total distance traveled. There was a main effect of Sex ($F_{(1,67)}=4.03$, $p<0.05$), but there were no effects of poly(I:C) ($F_{(1,67)}=0.01$, $p=0.92$) or THC ($F_{(1,67)}=0.008$, $p=0.93$) and no Poly(I:C) x THC interaction ($F_{(1,67)}=0.0001$, $p=0.99$). (**B**) Time that mice were immobile. There was no effect of Poly(I:C) ($F_{(1,67)}=1.29$, $p=0.26$) or THC ($F_{(1,67)}=0.012$, $p=0.91$), and there was no Poly(I:C) x THC interaction ($F_{(1,67)}=0.11$, $p=0.75$). (**C**) Number of immobile episodes while in the open field. There was no effect of Poly(I:C) ($F_{(1,67)}=0.32$, $p=0.57$) or THC ($F_{(1,67)}=0.12$, $p=0.73$), and there was no Poly(I:C) x THC interaction ($F_{(1,67)}<0.0001$, $p=0.99$). (**D**) Time spent in the inner zone of the open field. There was a main effect of Sex ($F(1,67)=12.75$, $p<0.001$), but there were no effects of Poly(I:C) ($F_{(1,67)}=0.24$, $p=0.62$) or THC ($F_{(1,67)}=0.90$, $p=0.35$) and no Poly(I:C) x THC interaction ($F_{(1,67)}=0.88$, $p=0.35$). All values are mean ± SEM.

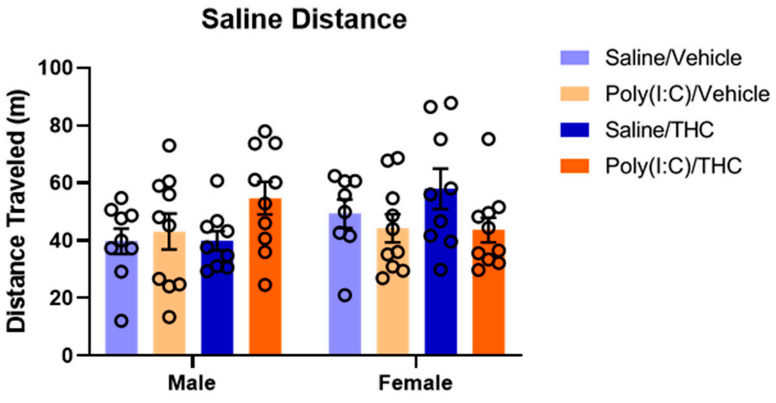

Figure S2. Distance traveled following saline administration in the amphetamine hyperlocomotion assay. Three-way ANOVA indicated that there were no significant main effects of Poly(I:C) ($F_{(1,67)}=0.007$, $p=0.93$), THC ($F_{(1,67)}=1.76$, $p=0.19$), or Sex ($F_{(1,67)}=1.42$, $p=0.24$). There was a significant Sex x Poly(I:C) interaction ($F_{(1,67)}=6.36$, $p<0.05$). *Post hoc* tests were not significant, but examination of the results indicates poly(I:C) treatment tended to increase locomotion in males whereas it tended to decrease locomotion in female mice. This effect may be due to differences in the effects of injection or due to difference in the time that mice took to acclimate to the novel open field. All values are mean ± SEM.

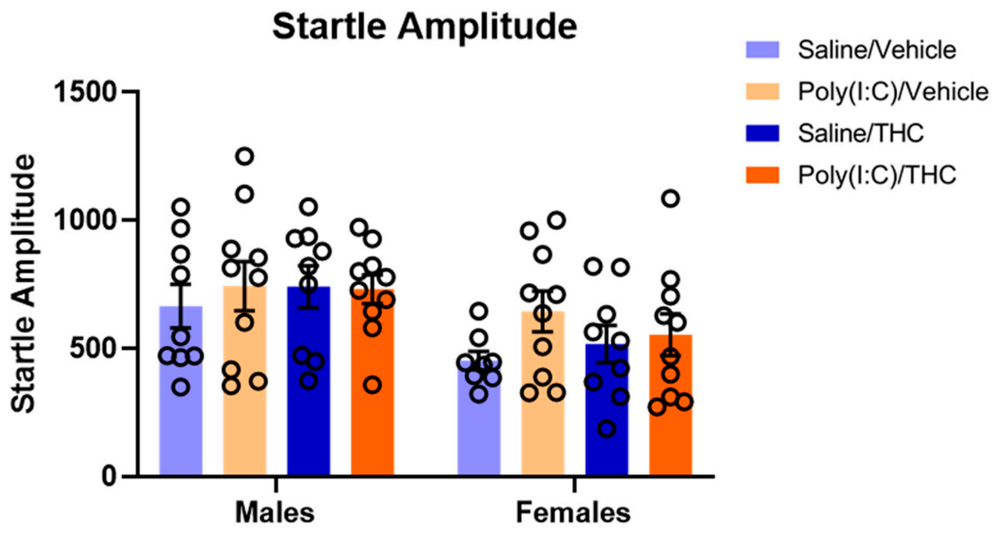

Figure S3. Startle amplitude in the PPI assay. There was a main effect of sex on startle amplitude ($F_{(1,67)}$=10.54, p<0.01). All values are mean ± SEM.

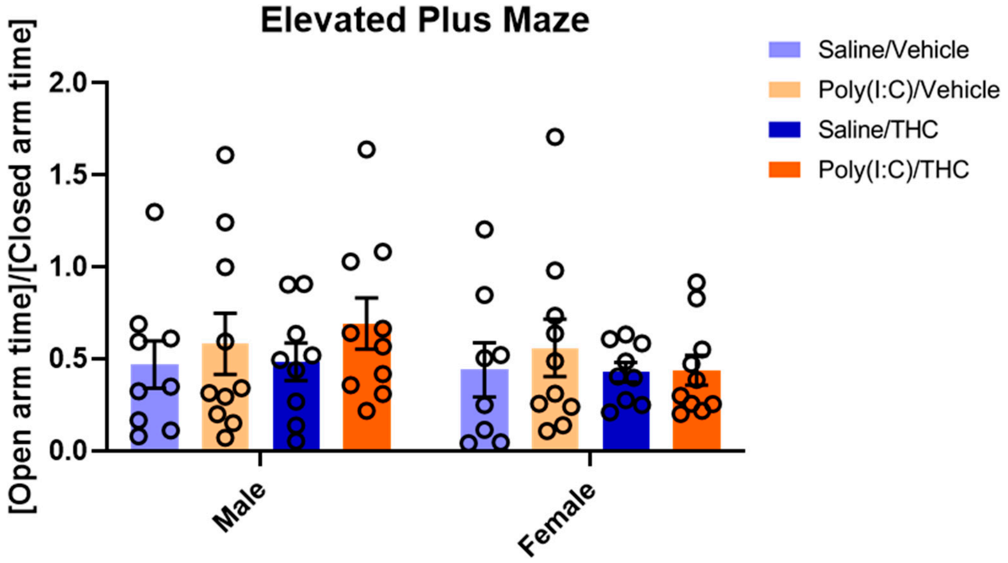

Figure S4. Elevated Plus Maze. The ratio of time spent in the open arms to the time spent in the closed arms was calculated. When analyzed by three-way ANOVA, there was no effect of Poly(I:C) ($F_{(1,67)}$=1.53, p=0.22) or THC ($F_{(1,67)}$=0.0007, p=0.98), and there was no Poly(I:C) x THC interaction ($F_{(1,67)}$=0.001, p=0.97). All values are mean ± SEM.

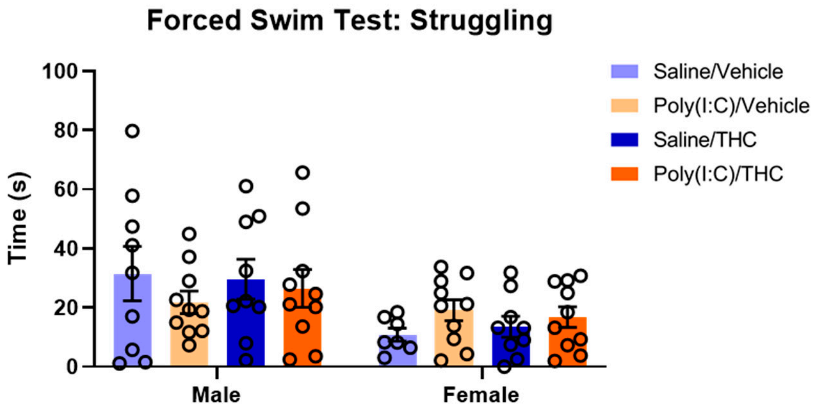

Figure S5. Forced Swim Test. When analyzed by three-way ANOVA, there was a main effect of Sex ($F_{(1,66)}$=10.34, p<0.01), but no effects of Poly(I:C) ($F_{(1,67)}$=0.007, p=0.93) or THC ($F_{(1,67)}$=0.04, p=0.84) and no Poly(I:C) x THC interaction ($F_{(1,67)}$=0.01, p=0.92). There was a trend towards a Sex x Poly(I:C) interaction ($F_{(1,66)}$=2.54, p=0.12). All values are mean ± SEM.
